# Can serum procalcitonin levels be useful in predicting spontaneous ureteral stone passage?

**DOI:** 10.1186/s12894-020-00608-3

**Published:** 2020-04-19

**Authors:** Nusret Can Cilesiz, Arif Ozkan, Arif Kalkanli, Ali Eroglu, Cem Tuğrul Gezmis, Berkan Simsek, Burak Arslan

**Affiliations:** 1 Department of Urology, GOP Taksim Education Training and Research Hospital, Karayolları Str. No:621 Gaziosmanpasa, İstanbul, Turkey; 2 Department of Urology, Yeniyüzyıl University Medicine Faculty, Gaziosmanpaşa Hospital, İstanbul, Turkey; 3 Bitlis State Hospital, Bitlis, Turkey 4 10 Sancaktepe State Hospital, İstanbul, Turkey; 4 Department of Urology, Sancaktepe State Hospital, İstanbul, Turkey

**Keywords:** Ureteral stone, Spontaneous stone passage, Medical expulsive therapy, Serum procalcitonin, Leucocyturia

## Abstract

**Background:**

Medical expulsive therapy (MET) is recommended for ureteral stones when there is no indication for interventional treatment. Spontaneous passage (SP) may not always be perceived in patients undergoing MET. We aimed to demonstrate the effects of inflammatory factors on spontaneous ureteral stone passage in patients undergoing MET.

**Methods:**

Our study was conducted between August and November, 2016, in healthy volunteers and patients with a single distal ureteral stone between 5 and 10 mm in diameter and no indications for interventional therapy. Blood and urine samples from all patients and healthy volunteers were tested. The patients were followed up every 2 weeks for 1 month unless emergency situations appeared. Patients with stone-free status at follow-up were concluded to have achieved complete stone passage [SP(+)], and failure [SP(−)] was concluded if the patient had not passed the stone by the end of the study. Blood samples of the patients and the control group were analyzed, recording WBC (white blood cell), CRP (c-reactive protein), SED (sedimentation), MPV (mean platelet volume), NLR (neutrophil-to-lymphocyte ratio), and serum procalcitonin levels. Abnormalities in urine samples were recorded. All patients received diclofenac sodium 75 mg/day, tamsulosin 0.4 mg/day, and at least 3 l/day fluid intake. Patients were followed for a month with kidney, ureter, bladder (KUB) plain films, ultrasonography (USG), and unenhanced abdominal CT scans while undergoing MET. Comparative statistical analyses were performed between the SP(+) and SP(−) groups.

**Results:**

The procalcitonin levels of the SP(−) group were significantly higher (207 ± 145.1 pg/ml) than in the SP(+) group (132.7 ± 28.1 pg/ml) (*p* = 0.000). The leucocyturia rate of the SP(−) group was significantly higher than in the SP(+) group (*p* = 0.004). Based on the ROC curve analysis, 160 pg/ml (86.7% sensitivity, 70.8% specificity, *p* < 0.001; AUC: 0.788 95% CI (0.658–0.917) was identified as the optimal cut-off value for procalcitonin. In logistic regression analysis, a significant efficacy of procalcitonin and leucocyturia was observed in the univariate analysis on spontaneous passage. In the multivariate analysis, significant independent activity was observed with procalcitonin. (*p* < 0.05).

**Conclusion:**

Our findings suggest that high procalcitonin levels and the presence of leucocyturia have a strong negative effect on SP of ureteral stones between 5 and 10 mm in diameter. This relationship can be explained by stone impaction, possibly caused by increased mucosal inflammation.

## Background

Urinary tract stones’ localization is usually on the kidney, while ureteral stones make up 20% of all urinary system stones [1]. Patients with a ureteral stone usually present with complaints such as flank pain, nausea-vomiting, and lower urinary tract symptoms [1, 2]. The most important parameter in determining the treatment to be applied is the size of the ureteral stones. Ureteral stones classified as non-complicated and smaller than 10 mm in diameter may be followed up for a month due to the possibility of spontaneous passage (SP).

Medical expulsive therapy (MET) is a non-invasive treatment modality that is especially recommended for ureteral stones > 5 mm. MET consists of high fluid intake and medical agents such as anti-inflammatory drugs, alpha-blockers, calcium channel blockers, corticosteroids or phosphodiesterase inhibitors. It has been proven elsewhere that the likelihood and speed of spontaneous passage are increased with MET [1, 3]. Furthermore, MET decreases hospital visits, costs, and the requirement of interventional treatment [4–7].

Ureteral stones can cause inflammation in the ureteral wall, and this mucosal inflammation was found to be related to the impaction of the stone. In studies using serum inflammation markers to predict the impaction of ureteral stones, serum white blood cell (WBC), neutrophil count, c-reactive protein (CRP), and neutrophil-lymphocyte ratio (NLR) values were found to be related to the spontaneous passage of ureteral stones [8–11].

Procalcitonin is a polypeptide consisting of 116 amino acids, with a molecular weight of approximately 13 kilodaltons. Ghillani et al., 1989, first described this hormone as the precursor of calcitonin, which is made up of 32 amino acids [12]. In sepsis and inflammation, serum procalcitonin levels have been shown to increase following release of endotoxins and immunomodulators [13]. Furthermore, it has been reported that the elevation of procalcitonin may be used to indicate a relapse of chronic prostatitis, the rate of infection in obstructed ureteral stones, and the severity of inflammation in cholecystitis [14–16].

Despite MET, the spontaneous passage of ureteral stones smaller than 10 mm may not be observed in clinical practice. In recent years, many studies have investigated the reasons behind this circumstance [10, 11]. In light of these studies, we aimed to investigate the effect of serum procalcitonin and other inflammation markers at the time of diagnosis, for patients with ureteral stones sized 5-10 mm.

## Methods

This prospective observational study was conducted between August and November of 2016, after receiving institutional review board approval. The patients gave informed consent for participation at the beginning of the study.

Patients aged 20 to 60 years with renal colic and having a single distal ureteral stone 5-10 mm in diameter, detected by an unenhanced CT scan, were included in this study. Exclusion criteria from the study were the patients with severe hydronephrosis, symptomatic urinary system infection, findings of acute renal failure, congenital ureteral anomaly, a history of ureteral stenosis or reconstructive ureteral surgery, and those who had received anti-inflammatory or anti-microbial drugs for the ureteral stone. Patients with active infective disease, chronic inflammatory disease (e.g., ulcerative colitis, rheumatoid arthritis, ankylosing spondylitis), active neoplasia, thyroid disease with or without thyroid surgery, immunosuppression or immunosuppressive treatment were also excluded from the study. The control group included 33 healthy volunteers of the same age range and socio-demographic characteristics, no history of stone disease and no active or chronic disease.

The patients’ characteristics including age, gender, BMI (body mass index), smoking history, chronic illnesses, drug allergies, previous stone interventions (nephropyelolithotomy, percutaneous surgery, URS-L or ESWL) were all recorded. Unenhanced abdominal CT scans were used to determine the stone size (dimension at its greatest diameter), area (width x length × 0.8), laterality (left/right), and the degree of hydronephrosis.

Complete blood cell count, C-reactive protein (CRP), sedimentation, serum procalcitonin, urinalysis and urine culture were performed for both groups. In the complete blood cell count, values for white blood cells (WBC), neutrophil/lymphocyte ratio (NLR), mean platelet volume (MPV), and red cell distribution width (RDW) were noted. In the urinalysis, the presence or absence of leucocyturia and bacteriuria were noted. The BioVendor® (Czechia) Human Procalcitonin ELISA (RD191006200R) kit was used to measure the procalcitonin level. Serums were analyzed using the Biotek Elx-800 microplate reader and Biotek Gen5 software.

All patients received diclofenac sodium 75 mg/day, tamsulosin 0.4 mg/day and at least 3 l/day fluid intake. The patients were followed up with kidney, ureter, bladder (KUB) plain films, ultrasonography (USG) and unenhanced abdominal CT scan every 2 weeks for 1 month unless emergency situations appeared. Patients with stone-free status at follow-up were concluded to have achieved complete stone passage [SP(+)], and failure [SP(−)] was concluded if the patient had not passed the stone by the end of the study.

### Statistical analyses

Mean, standard deviation, median, lowest and highest scores, frequency and ratio values were used for the descriptive statistics of the data. Distribution of variables was obtained using the Kolmogorov Smirnov test. The Mann-Whitney U test was utilized for analyses of the independent quantitative data. For the analyses of independent categorical data, the chi square test was preferred, and when it was not applicable, Fischer’s exact test was substituted. Impact level and cut-off values were determined using the ROC curve. The effect level was investigated by univariate and multivariate logistic regression. All analyses were conducted with SPSS 22.0; the accepted level of statistical significance was *p* < 0.05.

## Results

According to the exclusion criteria, 11 patients were excluded from the study, and the data of the case (*n* = 54) and control (*n* = 33) groups were analyzed. None of the patients showed symptoms requiring intervention treatment, such as high fever, renal colic attacks that cannot be controlled with analgesics, progressive hydronephrosis or renal failure, during the follow-up period.

There were no significant differences between the case and the control groups in terms of bacteriuria ratio, WBC or CRP values. However, the leucocyturia ratio, RDW, NLR, sedimentation and procalcitonin values were significantly higher in the case group (*p* < 0.05) (Table 1).

**Table 1:** Comparison of case and control groups’s sosyodemographic characteristics and biochemichal inflammation markers

	Case Group	Control Group	P
**Age (years)**	37,1 ± 10,2	33,9 ± 8,3	0,159
**Gender**	Male 38 (70,4%)	Male 27 (81,8%)	0,233
**BMI (kg/m**^**2**^**)**	26,5 ± 4,1	24,8 ± 5,1	0,245
**WBC (10**^**3**^**/**μL**)**	8,8 ± 2,3	8,0 ± 1,9	0,553
**RDW (%)**	15,5 ± 1,9	12,8 ± 0,9	**0,000**
**NLR (%)**	2,8 ± 1,7	2,0 ± 0,3	**0,009**
**MPV (fL)**	8,6 ± 1,0	8,5 ± 0,7	0,451
**CRP (mg/L)**	10,4 ± 12,6	4,0 ± 1,1	0,088
**Sedimentation (mm/h)**	13,4 ± 9,9	4,2 ± 1,3	**0,000**
**Procalcitonin (pg/ml)**	165,7 ± 104,7	128,2 ± 36,6	**0,004**
**Bacteriuria (+)**	3,7%	0%	0,524
**Leucocyturia (+)**	37%	0%	**0,000**

*BMI* Body mass index; *WBC* White blood cell; *NLR* Neutrophil-lymphocite ratio; *MPV* Mean platelet volume; *CRP* C-reactive protein

Based on the follow-up controls, 30 patients (55.5%) passed the stone [SP(+)], whereas passage did not occur in 24 patients (44.5%) [SP(−)]. Age, gender, BMI, smoking history, and previous stone-related intervention rates were similar in both groups except stone distributions (*p* < 0.05) (Table 2).

**Table 2:** Sosyodemographic characteristics and stone history of SP(−) and SP(+) groups

	SP(−)	SP(+)	p
**Age (years)**	35,8 ± 9,3	38,0 ± 10,9	0,381
**Gender (F/M) %**	41,7/58,3	20/80	0,083
**BMI (kg/m**^**2**^**)**	26,4 ± 4,7	26,5 ± 3,6	0,781
**Smoking history**	%50	%53,3	0,808
**Previous ESWL**	%12,5	%16,7	0,668
**Previous Stone surgery**	%8,3	%6,7	0,816
**Stone burden (mm**^**2**^**)**	30 ± 8.2	29.1 ± 10.3	0,310
**Stone distrubition (R/L)**	16/14	13/11	0,745

*BMI* Body mass index; *ESWL* Extracorporeal shock wave lithotripsy

Between the SP(−) and SP(+) groups, bacteriuria ratios, WBC, RDW, NLR, MPV, CRP, and sedimentation values were not significantly different (*p* > 0.05).

**Fig. 1:**
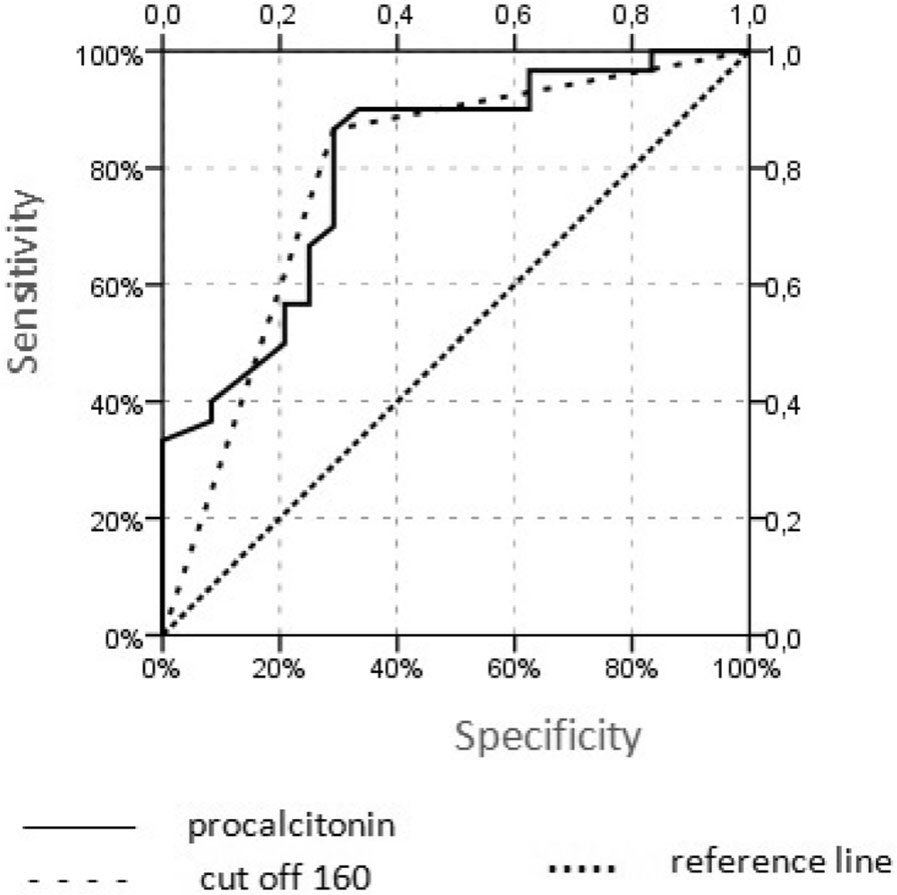
ROC curve for cutoff value of 160 pg/ml of procalcitonin on prediction of spontaneous ureteral stone passage

Compared to the SP (+) group, mean serum procalcitonin levels (207 ± 145.1 vs 132.7 ± 28.1 pg/ml, *p* = 0.000) and the leucocyturia ratio (58.3 vs. 20%, *p* = 0.004) were significantly higher in the SP (−) group (Table 3). Based on the ROC curve analysis, 160 pg/ml (86.7% sensitivity, 70.8% specificity, *p* < 0.001; AUC: 0.788 95% CI (0.658–0.917) was identified as the optimal cut-off value for procalcitonin (Fig. 1). In logistic regression analysis, a significant efficacy of procalcitonin and leucocyturia was observed in the univariate analysis of spontaneous passage. In the multivariate analysis, significant independent activity was observed with procalcitonin. (Table 4) (*p* < 0.05).

**Table 3:** Effect of biochemichal inflammation markers for spontaneous ureteral stone passage

	SP(−)	SP(+)	p
**WBC (10**^**3**^**/μL)**	8,4 + 2,3	9,1 + 2,4	0,261
**RDW (%)**	15,5 + 2,4	15,4 + 1,5	0,657
**NLR (%)**	2,8 + 1,7	2,9 + 1,7	0,821
**MPV (fL)**	8,7 + 0,6	8,5 + 1,3	0,721
**CRP (mg/L)**	9,0 + 8,2	11,5 + 15,4	0,875
**Sedimentation (mm/h)**	11,8 + 7	14,7 + 11,7	0,558
**Procalcitonin (pg/ml)**	207 + 145,1	132,7 + 28,1	**0,000**
**Leucocyturia (+)(%)**	58,3%	20%	**0,004**

*WBC* White blood cell; *NLR* Neutrophil-lymphocite ratio; *MPV* Mean platelet volume; *CRP* C-reactive protein

**Table 4:** Logistic regression analysis for spontaneous passage in 5-10 mm distal ureteral stones

	Univariate analysis			Multivariate analysis		
	OR	95% Confidence Interval	p	OR	95% Confidence Interval	p
**Age (years)**	1,02	0,97-1,08	0,447			
**Gender**	2,86	0,85-9,56	0,088			
**BMI**	1,00	0,88–1,15	0,962			
**Primary/secondary**	1,27	0,43-3,82	0,665			
**History of SWL**	0,96	0,32-2,93	0,950			
**History of stone passage**	1,40	0,30-6,56	0,669			
**History of stone surgery**	0,79	0,10-6,03	0,817			
**Smoking history**	1,14	0,39-3,35	0,808			
**Hydronephrosis**	0,58	0,19-1,72	0,326			
**Stone burden**	0,99	0,95-1,04	0,732			
**WBC**	1,14	0,89-1,46	0,287			
**RDW**	0,97	0,73-1,28	0,807			
**NLR**	1,03	0,74-1,43	0,855			
**MPV**	0,84	0,47-1,48	0,547			
**CRP**	1,02	0,97-1,07	0,470			
**Sedimentation**	1,03	0,97-1,10	0,307			
**Procalcitonin**	0,96	0,94-0,99	***0,002***	0,94	0,89- 0,98	***0,005***
**Leucocyturia**	0,18	0,05-0,60	***0,005***			

*BMI* Body mass index; *ESWL* Extracorporeal shock wave lithotripsy; *WBC* White blood cell; *NLR* Neutrophil-lymphocite ratio; *MPV* Mean platelet volume; *CRP* C-reactive protein

## Discussion

Observation with MET protocol, ESWL and URS-L are the treatment options for the ureteral stones and could be preferred based on the patient’s clinical condition and size of the stone. Treatment success for ESWL and URS-L depends on localization and size of the stone and is reported to be 68–90% and 80–97% respectively [1]. Even though high success rates occur from these treatment modalities, high costs and complication risks must be weighed as to their primary disadvantages [17, 18]. Moreover, the financial burden of having additional laboratory tests is another controversy about whether a ureteral stone can be spontaneously evacuated or not [18]. Similarly, complications such as recurrent renal colic attacks, urosepsis, and urinary tract infections may occur; observation or MET is therefore to be preferred for those patients. During the observation, ureteral stone impaction may also be observed due to ureteral mucosal inflammation, resulting in a more difficult and complicated treatment process of the ureteral stone [11]. For these reasons, accurate prediction of spontaneous passage has taken on greater importance in recent years, and several studies have been conducted in this area.

Recent studies have determined the likelihood of spontaneous passage in ureteral stones < 5 mm to be 71–100%, and 25–46% in stones measuring 5-10 mm. Also, for ureteral stones < 4 mm, the possibility of spontaneous passage within 40 days was reported to be 95% [1, 19]. One study classified stones into three groups based on size and reported spontaneous passage rates of 89.9, 63.4 and 9.1% for < 5 mm, 5-10 mm and > 10 mm ureteral stones, respectively [20]. In another study, which compared observation and MET for 5-10 mm ureteral stones, SP ratios were reported to be 50 and 81.8% respectively [21]. In our study, when medical expulsive therapy was conducted for the 5-10 mm ureteral stones, the SP rate was calculated to be 55.5%.

Another important factor affecting the possibility of SP in ureteral stones is localization. Studies indicate that as the stone approaches to distal, both the SP ratio and the greatest benefit from MET increase [22–24]. Although MET was employed in our study, the SP rate was lower than in other studies. AUA panels showed superior SFR for patients treated with MET (77.3%) compared with placebo (54.4%) on distal ureteral stones < 10 mm [22]. We believe that the lower rate of SFR (55.5%) in our study was due to the 5-10 mm stone size in the patients in our research.

Lifestyle factors, such as smoking habits and sexual intercourse, have also been investigated in recent years as potential factors of spontaneous passage. Fazlıoğlu et al. reported similar SP ratios in < 4 mm distal ureter stones for smokers and non-smokers, but for ≥ 4 mm distal ureter stones, the SP ratio was reported to be lower in smokers [25]. In another study, Bayraktar et al. investigated three groups of patients with 5-10 mm distal ureteral stones receiving different treatments. Group 1 received tamsulosin 0.4 mg/day, Group 2 was asked to have intercourse three times per week, and Group 3 received medical treatment. The authors reported that, based on their results, having sexual intercourse three times a week could be as beneficial as tamsulosin treatment [26]. We also compared SP levels in terms of BMI and smoking habits, but the differences were not significant (*p* > 0.05).

In recent years, the effect of some biochemical factors on the SP ratio has been reported. Ahmed et al. reported that patients with small stones, distal localization, high serum WBC level, low perinephric fat thickness, and lack of tissue-rim sign had increased SP ratios [27]. Similarly, another study reported significant correlations between the increase of WBC and neutrophil levels and the SP ratio, in < 10 mm ureteral stones (*p* < 0.001) [11]. According to the authors, this correlation was associated with increased ureteral peristalsis, caused by inflammation in the ureteral wall. By contrast, Aldaqodossi et al. reported that an increase in ureteral inflammation could decrease the SP ratio [8]. In this study, the authors reported that the SP(−) group was significantly higher than the SP(+) group for initial CRP values (*p* = 0.001). Furthermore, based on ROC curve analysis, it was found that sensitivity and specificity of the 21.9 mg/dl cut-off value for CRP, were 78.6 and 89.3% respectively [8]. In our study, the mean serum WBC value was higher in the SP(+) group, when compared to the SP(−) group (9.1 ± 2.4 vs. 8.4 ± 2.3), although this difference is not statistically significant. Similarly, mean CRP values were higher in the SP(+) group (11.5 ± 15.4) than in the SP(−) group (9.0 ± 8.2), and again, there is no statistically significant difference.

To our knowledge, the available literature does not include a study investigating the effect of serum procalcitonin on SP. However, procalcitonin has been reported to be valuable in showing a correlation between urinary tract infection and impacted ureteral stones. Papagiannopoulos et al. reported that procalcitonin > 100 pg/ml was detected in 18% of patients treated with MET, 45% of whom had undergone URS-L or ureteral stenting [16]. In our study, the mean serum procalcitonin level of the SP(−) group (207.0 ± 145.1 pg/ml) was significantly higher than in the SP(+) group (132.7 ± 28.1 pg/ml) (*p* = 0.000). Furthermore, leucocyturia rates of the SP(−) group were significantly higher than in the SP(+) group (58.3% vs. 20%) (*p* = 0.004). This finding could better explain the relationship between high serum procalcitonin and mucosal inflammation. It has been reported in previous studies that leucocyturia did not effect on spontaneous passage; this finding should be re-evaluated through studies with larger sample sizes [9, 27].

The fact that procalcitonin is significant even in multivariant analysis increases the possibility of using this marker in daily practice in the future. Of course, the accuracy of this information should be supported by a larger series.

Based on these results, it could be argued that procalcitonin plays a role in predicting SP, but this hypothesis needs to be supported with studies having higher sample sizes. Also, measuring the procalcitonin level again at the end of the study could increase the strength of the study. Our sample size was not large enough for subgroup analysis, a limitation of our study. Particularly, evaluating the serum procalcitonin and leucocyturia through subgroup analysis may provide a more objective argument for our hypothesis.

## Conclusion

This study reports that distal ureteral stones sized 5-10 mm presenting with high procalcitonin levels and leucocyturia may have a negative effect on spontaneous passage. This relationship can be explained by stone impaction, possibly caused by increased mucosal inflammation.

## Data Availability

All data generated or analysed during this study are included in this published article and its supplementary information files. If someone wants to access the data of the study, they can contact the corresponding author (nusretcancilesiz@gmail.com).

## Abbreviations

MET: Medical expulsive therapy
SP: Spontaneous passage
KUB: Kidney, ureter, bladder plain films
USG: Ultrasonography
CT: Computerized tomography
URS-L: Ureterorenoscopic lithotripsy
ESWL: Extracorporeal shock wave lithotripsy
VAS: Visual Analogue Scale
BMI: Body mass index
AUA: American Urological Association

## References

[1] Türk C, Petřík A, Sarica K, Seitz C, Skolarikos A, Straub M (2016). EAU guidelines on diagnosis and conservative Management of Urolithiasis. Eur Urol.

[2] Pak CY. Kidney stones. Lancet. 1998;351(9118):1797–1801. Review. 10.1016/S0140-6736(98)01295-1 9635968

[3] Ordon M, Andonian S, Blew B, Schuler T, Chew B (2015). Pace KT CUA, guideline: management of ureteral calculi. Can Urol Assoc J.

[4] Singh A, Alter HJ, Littlepage A (2017). A systematic review of medical therapy to facilitate passage of ureteral calculi. Ann Emerg Med.

[5] Loftus C, Nyame Y, Hinck B, Greene D, Chaparala H, Alazem K (2016). Medical expulsive therapy is underused for the management of renal colic in the emergency setting. J Urol.

[6] Brede C, Hollingsworth JM, Faerber GJ, Taylor JS, Wolf JS (2010). Medical expulsive therapy for ureteral calculi in the real world: targeted education increases use and improves patient outcome. J Urol.

[7] Furyk JS, Chu K, Banks C, Greenslade J, Keijzers G, Thom O et al Distal Ureteric Stones and Tamsulosin: A Double-Blind, Placebo-Controlled, Randomized, Multicenter Trial. Ann Emerg Med. 2016;67(1):86–95.e2. 10.1016/j.annemergmed.2015.06.001 26194935

[8] Aldaqadossi HA (2013). Stone expulsion rate of small distal ureteric calculi could be predicted with plasma C-reactive protein. Urolithiasis.

[9] Lee KS, Ha JS, Koo KC (2017). Significance of neutrophil-to-lymphocyte ratio as a novel Indicator of spontaneous ureter stone passage. Yonsei Med J.

[10] Özcan C, Aydoğdu O, Senocak C, Damar E, Eraslan A, Oztuna D (2015). Predictive factors for spontaneous stone passage and the potential role of serum C-reactive protein in patients with 4 to 10 mm distal ureteral stones: a prospective clinical study. J Urol.

[11] Sfoungaristos S, Kavouras A, Katafigiotis I, Perimenis P (2012). Role of white blood cell and neutrophil counts in predicting spontaneous stone passage in patients with renal colic. BJU Int.

[12] Aouifi A, Piriou V, Blanc P (1999). Effect of cardiopulmonary bypass on serum procalcitonin and C-reactive protein concentrations. Br J Anaesth.

[13] Meisner M. Procalcitonin: a new innovative infection parameter. In: Meisner M, ed. Biochemistry. Stuttgart: Brahms Diagnostica; 2000: 15.

[14] Çakıroğlu B, Eyyüpoğlu E, Balcı MBC, Hazar Aİ, Uyanık BS, Doğan AN (2016). The evaluation of the procalcitonin levels in chronic prostatitis patients. Nobel Med.

[15] Yuzbasioglu Y, Duymaz H, Tanrikulu CS, Halhalli HC, Koc MO, Tandoğan M (2016). Role of Procalcitonin in evaluation of the severity of acute Cholecystitis. Eurasian J Med.

[16] Papagiannopoulos D, Whelan P, Ahmad W, Rybak J, Hota B, Deane L (2016). Procalcitonin is a strong predictor of urine culture results in patients with obstructing ureteral stones: a prospective, pilot study. Urol Ann.

[17] Skolarikos A, Alivizatos G, de la Rosette J (2006). Extracorporeal shock wave lithotripsy 25 years later: complications and their prevention. Eur Urol.

[18] Perez Castro E, Osther PJ, Jinga V, Razvi H, Stravodimos KG, Parikh K (2014). CROES Ureteroscopy global study group. Differences in ureteroscopic stone treatment and outcomes for distal, mid-, proximal, or multiple ureteral locations: the clinical research Office of the Endourological Society ureteroscopy global study. Eur Urol.

[19] Skolarikos A, Laguna MP, Alivizatos G, Kural AR, de la Rosette JJ (2010). The role for active monitoring in urinary stones: a systematic review. J Endourol.

[20] Demehri S, Steigner ML, Sodickson AD, Houseman EA, Rybicki FJ, Silverman SG (2012). CT-based determination of maximum ureteral stone area: a predictor of spontaneous passage. AJR Am J Roentgenol.

[21] Chau LH, Tai DC, Fung BT, Li JC, Fan CW, Li MK (2011). Medical expulsive therapy using alfuzosin for patient presenting with ureteral stone less than 10mm: a prospective randomized controlled trial. Int J Urol.

[22] Türk C, Knoll T, Seitz C, Skolarikos A, Chapple C, McClinton S;European Association of Urology. Medical expulsive therapy for Ureterolithiasis: the EAU recommendations in 2016. Eur Urol 2017;71(4):504–507. doi:10.1016/j.eururo.2016.07.024. Epub 2016 Aug 6. PubMed PMID: 27506951. 10.1016/j.eururo.2016.07.024 27506951

[23] Brubaker WD, Dallas KB, Elliott CS, Pao AC, Chertow GM, Leppert JT, Conti SL (2019). Payer type, race/ethnicity, and the timing of surgical Management of Urinary Stone Disease. J Endourol.

[24] Matlaga BR, Jansen JP, Meckley LM, Byrne TW, Lingeman JE (2012). Treatment of ureteral and renal stones: a systematic review and meta-analysis of randomized, controlled trials. J Urol.

[25] Fazlioglu A, Salman Y, Tandogdu Z, Kurtulus FO, Bas S, Cek M (2014). The effect of smoking on spontaneous passage of distal ureteral stones. BMC Urol.

[26] Bayraktar Z, Albayrak S (2017). Sexual intercourse as a new option in the medical expulsive therapy of distal ureteral stones in males: a prospective, randomized, controlled study. Int Urol Nephrol.

[27] Ahmed AF, Gabr AH, Emara AA, Ali M, Abdel-Aziz AS, Alshahrani S. Factors predicting the spontaneous passage of a ureteric calculus of ⩽10 mm. Arab J Urol. 2015;13(2):84–90. 10.1016/j.aju.2014.11.004 PMC4561928 26413326

